# Supplementary material for: Learning curve and surgical outcome of robotic assisted colorectal surgery with ERAS program
Source: Sci Rep. 2022 Nov 29;12:20566. doi: 10.1038/s41598-022-24665-w (PMC9709162; doi:10.1038/s41598-022-24665-w)
Supplement: Supplementary file 2 — Supplementary Information 2. [file 41598_2022_24665_MOESM2_ESM.zip › RAL Raw data-Ñ[▒K/098/Patho_ñw╝╨░O▒Kñσ.pdf]

臺中榮民總醫院 臺中市臺灣大道四段1650號 病理部活體組織切片 臨床醫學實驗及病理診斷中心 檢驗(查)報告

[redacted]

Pathologic diagnosis:
Rectum + vagina, robotic assisted wedge resection ---
Gastrointestinal stromal tumor of low risk. Tumor involved wall of rectum.

Ancillary study for diagnosis:
CD117 (+) stain done on section C.

Prognostic and predictive factor:
1. Tumor site: Specify: rectum.
2. Tumor size: 40x35 mm.
3. Tumor focality: Unifocal.
4. GIST subtype: Spindle cell.
5. Mitotic rate: <1 /5 mm2 (approximately 20 to 25 HPF. See Note).
6. Necrosis: Not identified.
7. Histologic grade: G1: Low grade; mitotic rate ≦5/ 5 mm2.
8. Margins: Involved by GIST, specify margin: posterior soft tissue margin.
9. Risk assessment: Low risk.
10. Regional Lymph Nodes: No lymph nodes submitted or found.
11. Distant metastasis: Cannot be assessed.
12. Prebiopsy treatment (select all that apply): No therapy.
13. Treatment effect: No known presurgical therapy.
14. Pathological TNM stage: pT2Nx (According to the eighth edition, American Joint Committee on Cancer Staging Guidelines for Tumors).
15. TNM descriptors: Not applicable.

Gross description:
The specimen consists of a piece of tan soft tissue, 4.5x4x2 cm, with an attached rectal mucosa, 2x1 cm, and an attached vaginal mucosa, 2x2 cm. There is a gray white firm tumor, 4x3.5 cm, located over the wall of recum. The posterior margin is very close to tumor. Representative parts for section: A) right margin B) left margin C-J) tumor (yellow ink: rectal side) (mapping: D+E, F+G, H+J).

Note:
1. The type of specimen fixation and processing: Formalin-fixed paraffin-embedded sections.
2. The antibody clone(vendor) and detection system used: CD117: polyclonal rabbit(DAKO)/VENTANA, CD34: monoclonal mouse(DAKO)/VENTANA. Type of method: LSAB.
3. The test is fixed in 10% neutral buffered formalin for at least 6 hours and no longer 24 hours.
4. The required total count of mitoses is per 5 mm2 on the glass slide section. With the use of older model microscopes, 50 HPF is equivalent to 5 mm 2. Most modern microscopes with wider 40X lenses/fields require approximately 20 to 25 HPF to encompass 5 mm2.
#T-59600_2 #R-1030A_2 9 1104 S00000

已電子簽章

敬告：本單僅供醫師參考使用，不具任何申請效力！！
請病患妥為保管，若不慎遺失需自負個人資料外洩責任！
